# Wogonin Inhibits Cardiac Hypertrophy by Activating Nrf-2-Mediated Antioxidant Responses

**DOI:** 10.1155/2021/9995342

**Published:** 2021-07-01

**Authors:** Xiaowen Shi, Bin Zhang, Zhenliang Chu, Bingjiang Han, Xueping Zhang, Ping Huang, Jibo Han

**Affiliations:** Department of Cardiology, The Second Affiliated Hospital of Jiaxing University, Jiaxing, Zhejiang 314000, China

## Abstract

**Background:**

Cardiac hypertrophy is one of the initial disorders of the cardiovascular system and can induce heart failure. Oxidative stress is an important pathophysiological mechanism of cardiac hypertrophy. Wogonin (Wog), an important flavonoid derived from the root of *Scutellaria baicalensis Georgi*, is known to possess antioxidant properties.

**Methods:**

An *in vitro* model of cardiac hypertrophy was established by stimulating H9C2 cells and neonatal rat cardiomyocytes (NRCMs) with angiotensin II (AngII). The indices related to myocardial hypertrophy and oxidative stress were detected. An *in vivo* model of cardiac hypertrophy was induced by transverse aortic constriction (TAC) in C57BL/6 mice. Cardiac function was monitored by chest echocardiography, and the hypertrophy index was measured. The mice were then sacrificed for histological assays, with mRNA and protein detection. To further explore the role of nuclear factor- (erythroid-derived 2-) like 2 (Nrf-2) in regulating the antioxidant effects of Wog in cardiac hypertrophy, siRNA analysis was conducted.

**Results:**

Our results showed that Wog significantly ameliorated AngII-induced cardiomyocyte hypertrophy by inhibiting oxidative stress in H9C2 cells and NRCMs. In addition, Wog treatment prevented oxidative stress and improved cardiac hypertrophy in mice that underwent TAC. Using gene-specific siRNA for *Nrf-2*, we discovered that these antioxidative effects of Wog are mediated through Nrf-2 induction.

**Conclusions:**

Our results provide further evidence for the potential use of Wog as an antioxidative agent for treatment of cardiac hypertrophy, and Nrf-2 might serve as a therapeutic target in the treatment of cardiac hypertrophy.

## 1. Introduction

Cardiac hypertrophy is the abnormal enlargement of the heart muscle and a severe disorder of the cardiovascular system. It is an adaptive response of the heart to virtually all forms of cardiac disease including hypertension, mechanical load, myocardial infarction, and cardiac arrhythmias [1, 2]. Many previous studies have indicated that dysregulated angiotensin II (AngII), the principal molecule of the renin-angiotensin system, plays an important role in the development of cardiac hypertrophy [3, 4]. However, better understanding of the pathogenesis of cardiac hypertrophy and the investigation of essential therapeutic methods are urgently required.

AngII plays a major role in the regulation of vascular function and the structure and has been established that contributes to the pathogenesis of cardiovascular diseases by its induced oxidative stress [5, 6]. Through stimulation of its type 1 receptor, AngII was shown to be associated with an overexpression of cytosolic proteins involved in the activation of the NAD(P)H oxidase, leading to cardiac dysfunction and cell hypertrophy [7]. In our study, an *in vitro* model of cardiac hypertrophy was created by stimulating rat myocardial cells (H9C2) with AngII (1 *μ*m). The crucial role of oxidative stress in cardiovascular diseases suggests that molecules with antioxidant properties may improve treatment efficacy.

Wogonin (5,7-dihydroxy-8-methoxyflavone (Wog)) is an important flavonoid derived from the root of *Scutellaria baicalensis Georgi* [8]. It has a wide range of pharmacological activities including antioxidant [9], anti-inflammatory [10], antitumor [11], and cardiovascular protection [12, 13]. However, whether and how Wog attenuates cardiac hypertrophy is unclear. Many of the protective effects of Wog are largely dependent on its antioxidant properties [14–16]. Therefore, we speculated that the antioxidant-like effect of Wog is a likely key to prevent cardiac hypertrophy.

Nuclear factor- (erythroid-derived 2-) like 2 (Nrf-2) is a regulator of the antioxidant system and upregulates the expression of heme oxygenase-1 (HO-1) and NADPH quinine oxidoreductase-1 (NQO-1) to reduce oxidative stress [17]. Nrf-2 is considered an important potential target for treating certain cardiovascular diseases such as cardiac hypertrophy [18, 19]. In this study, we aimed to determine whether Wog is protective against cardiac hypertrophy and explored the underlying mechanisms. Experimental results using AngII-induced hypertrophic cardiomyocytes confirmed that Wog suppressed oxidative stress via Nrf-2-mediated antioxidant responses to ameliorate cardiomyocyte hypertrophy. Our in vivo data showed that mice with transverse aortic constriction- (TAC-) induced hypertrophy had an increased heart size, ratio of heart weight to body weight, and oxidative stress levels, which were suppressed by Wog. Furthermore, using specific siRNA for *Nrf-2*, we detected that Wog protects against hypertrophic cardiomyocytes via Nrf-2-mediated antioxidant responses.

## 2. Materials and Methods

### 2.1. Materials

AngII was purchased from Sigma-Aldrich (St. Louis, MO, USA). Wog was purchased from Nanjing Spring & Autumn Biological Engineering (Nanjing, China) and dissolved in dimethyl sulfoxide for in vitro experiments. Anti-myosin heavy chain-beta (MyHC-*β*) and anti-Nrf-2 were obtained from Abcam (Cambridge, MA, USA). Anti-Keap-1, anti-Tubulin, anti-GAPDH, and secondary antibodies were purchased from Cell Signaling Technology (Danvers, MA, USA). Dihydroethidium (DHE) staining, malondialdehyde (MDA) kit, and superoxide dismutase (SOD) kit were purchased from Beyotime Institute of Biotechnology (Haimen, China). Rhodamine phalloidin was obtained from SolarBio (Beijing, China). Real-time RT-PCR reagents were purchased from TaKaRa Company (Dalian, China). All cell culture reagents were purchased from Gibco (Carlsbad, CA, USA).

### 2.2. Animal Experiments

C57BL/6 mice were obtained from the Jiaxing University Animal Centre. The animals were housed at 18–22°C room temperature with a relative humidity of 40–60%, with normal lighting conditions and fed with a standard rodent diet. All animal care and experimental procedures were performed in accordance with the Guidelines for the Care and Use of Laboratory Animals (US National Institutes of Health). Animal care and experimental protocols were approved by Council of Animal Care and Use of the Second Affiliated Hospital of Jiaxing University (Jiaxing, China; approval no. JXEY-2020JX061). Mice were randomly assigned into four groups (*n* = 5/group) as follows: sham, Wog, TAC, and TAC + Wog. The TAC operation procedures were performed as described previously [20, 21]. The sham-operated group underwent the same procedure without aortic ligation. Mice treated with Wog (single intragastric administration of Wog (10 mg/kg/day)) [22, 23]. Eight weeks after TAC, cardiac functions of the mice were assessed by echocardiography, with a Vevo 2100 High-Resolution Imaging System (VisualSonics Inc.) under 1% isoflurane anesthesia. After echocardiographic measurement, mice were sacrificed with the intraperitoneal injection of an overdose of sodium pentobarbital (200 mg/kg) and the hearts were collected for further evaluation.

### 2.3. Cell Culture and Treatment

Rat cardiomyocytes (H9C2) were obtained from Shanghai Institute for Biological Sciences, Chinese Academy of Sciences, and cultured in high-glucose DMEM and 10% heat-inactivated fetal bovine serum (FBS) supplemented with 100 U/mL penicillin and 100 g/mL streptomycin, at 37°C in a 95% air/5% CO_2_ incubator. Cells were transfected with siRNA using the Lipofectamine 2000 reagent (Invitrogen, Carlsbad, CA, USA). Gene-specific siRNA (5′-GGGUAAGUCGAGAAGUGUUTT-3′) for *Nrf-2* and negative control siRNA were purchased from GenePharma (Shanghai, China). The transfection efficiency of RNA knockdown was estimated 48 h posttransfection by Western blotting.

Neonatal rat cardiomyocytes (NRCMs) were isolated from Sprague-Dawley (SD) rats as described previously [24]. Briefly, rat hearts were cut into pieces, washed with ice-cold hanks balanced salt solution (HBSS) three times, and incubated with 0.125% trypsin-EDTA for 15 minutes at 34°C for a total of five times. Then, the NRCMs were centrifuged via a differential attachment technique then seeded in six-well culture plates at a density of 2 × 10^5^ cells per well. The isolated NRCMs were grown in DMEM containing 15% FBS supplemented with 100 U/mL penicillin and 100 g/mL streptomycin, at 37°C in a 95% air/5% CO_2_ incubator.

### 2.4. Cell Viability Assay

Cell viability was determined by CCK8 cytotoxicity assay. Briefly, cells were seeded in 96-well plates at 5000 cells/well and treated with Wog at different concentrations (1, 2.5, 5, 10, 20, 40, 60, and 80 *μ*M) for 48 h. Cells were incubated with CCK8 (10 *μ*L/well) for 1–4 h at 37°C and then spectrophotometrically quantified at 450–490 nm. Cell viability was calculated as follows: cell viability = *A*_treated/_*A*_control_ × 100%.

### 2.5. Phalloidin Staining of F-Actin Filaments

Briefly, cells were fixed with 4% paraformaldehyde, permeated with 0.1% Triton X-100, and stained with FITC-labeled phalloidin at a concentration of 100 nM for 30 min. The nuclei were stained in Hoechst solution at a concentration of 1 *μ*g/mL for 10 min. Immunofluorescence was captured using a fluorescence microscope (Axio Imager, Germany). The cell surface area was determined after cell staining and imaging by microscopy. The surface area was quantified by imaging to the complete boundary of 30 individual cells/condition using ImageJ software (National Institutes of Health).

### 2.6. Intracellular Reactive Oxygen Species Measurement

Dihydroethidium (DHE) staining was used to measure intracellular superoxide anion levels. Cells were exposed to AngII with or without Wog pretreatment. Cultured cells were incubated with 10 *μ*M DHE for 30 min. Cells were observed by fluorescence microscopy.

### 2.7. Measurement of Malondialdehyde and Superoxide Dismutase Levels

The activities of SOD and MDA were determined using commercially available kits according to the manufacturer's instructions (Beyotime Institute of Biotechnology, Haimen, China). In brief, cells were seeded into 6-well plates and treated as described above. Then medium was discarded, cells were collected into tubes, extraction reagent was added at a ratio of 1 ml reagent/1 × 10^7^ cells. For detection of the activities of MDA, with addition of other reagents, the mixture was stored at 100°C for 15 min and centrifuged at 10000 g for 10 min after cooling. Detection was performed at 450 nm. The activity of MDA was expressed as nmol/mg protein. To detect SOD activity, the mixture was placed in a water bath at 37°C for 30 min for measuring the absorbance value at 450 nm. The activity of SOD was expressed as U/mL.

### 2.8. Real-Time Quantitative Polymerase Chain Reaction

Total RNA was extracted from heart tissues and H9C2 cells using TRIzol reagent (Invitrogen) according to the manufacturer's instructions. Real-time RT-PCR was performed according to the instructions on the PCR kit (TaKaRa). Mastercycler (Eppendorf, Hamburg, Germany) was used for qPCR analysis. Primers were obtained from Thermo Fisher Scientific (primer sequences are listed in Table 1). Target mRNA was normalized to *β*-actin.

### 2.9. Western Blot Analysis

Heart tissues and H9C2 cells were homogenized in RIPA lysis buffer (Beyotime Institute of Biotechnology, Haimen, China) for 30 min on ice. Protein concentration was calculated using the bicinchoninic acid (BCA) method. Proteins (30 *μ*g) were separated by sodium dodecyl sulfate-polyacrylamide gel electrophoresis (SDS-PAGE) and transferred to polyvinylidene difluoride (PVDF) membranes. Each membrane was blocked for 1.5 h with 3% bovine serum albumin (BSA) at room temperature and incubated with primary antibodies overnight at 4°C. After washing, the membranes were incubated with HRP-conjugated secondary antibody and visualized using enhanced chemiluminescence (Bio-Rad). Protein quantities were analyzed using ImageJ software (version 1.38e) and normalized to their respective controls.

### 2.10. Statistical Analysis

Experiments were randomized and blinded. Data were presented as means ± SEM, and individual data points were plotted in figures. Statistical significance between groups was determined by the unpaired Student's *t*-test or one-way ANOVA in GraphPad Pro5.0 (GraphPad, San Diego, CA, USA). *P* < 0.05 was considered to indicate statistical significance.

## 3. Results

### 3.1. Wog Prevented AngII-Induced Cardiomyocyte Hypertrophy

**Figure 1:**
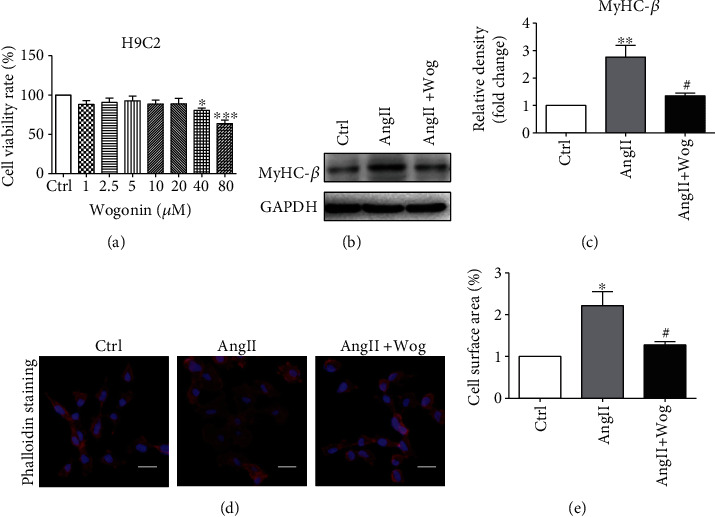
Wog prevented AngII-induced cardiomyocyte hypertrophy. H9C2 cells were pretreated with Wog (20 *μ*M) for 2 hours and then incubated with AngII (1 *μ*M) for the times indicated. (a) H9C2 cells were treated with Wog at the indicated concentrations for 48 h, and the potential effect of Wog treatment was assessed using the CCK8 cell viability assay (*n* = 6); (b, c) Western blot analysis of MyHC-*β* in H9C2 cells (*n* = 3); (d, e) representative images of rhodamine-labeled phalloidin staining from H9C2 cells (magnification: ×200, scale bar: 20 *μ*m). ^∗^*P* < 0.05, ^∗∗^*P* < 0.01, and ^∗∗∗^*P* < 0.001 compared to Ctrl; ^#^*P* < 0.05, ^##^*P* < 0.01, and ^###^*P* < 0.001 compared to AngII.

We first determined the viability of H9C2 cells following treatment with Wog. H9C2 cells were treated with various doses of Wog (1–80 *μ*M) for 48 h, and cell viability was assessed using the CCK8 viability assay. Wog treatment reduced cell viability only when used at 40 or 80 *μ*M (Figure 1(a)). Based on these results, we selected 20 *μ*M Wog to determine the effect on AngII-induced cellular damage.

AngII was used to establish the in vitro model of cardiac hypertrophy in this study [25, 26]. As shown in Figures 1(b) and 1(c) and Supplementary Figure 1A, AngII caused increased expression of the hypertrophic marker MyHC-*β* in H9C2 cells and NRCMs. However, Wog could attenuate the expression of MyHC-*β*, suggesting that it inhibited cardiomyocyte hypertrophy. Further, Wog reduced AngII-induced cell size increase, which was confirmed by rhodamine phalloidin staining (Figures 1(d) and 1(e) and Supplementary Figure 1B and 1D).

### 3.2. Wog Inhibited Oxidative Stress in Ang II-Induced Cardiomyocyte

**Figure 2:**
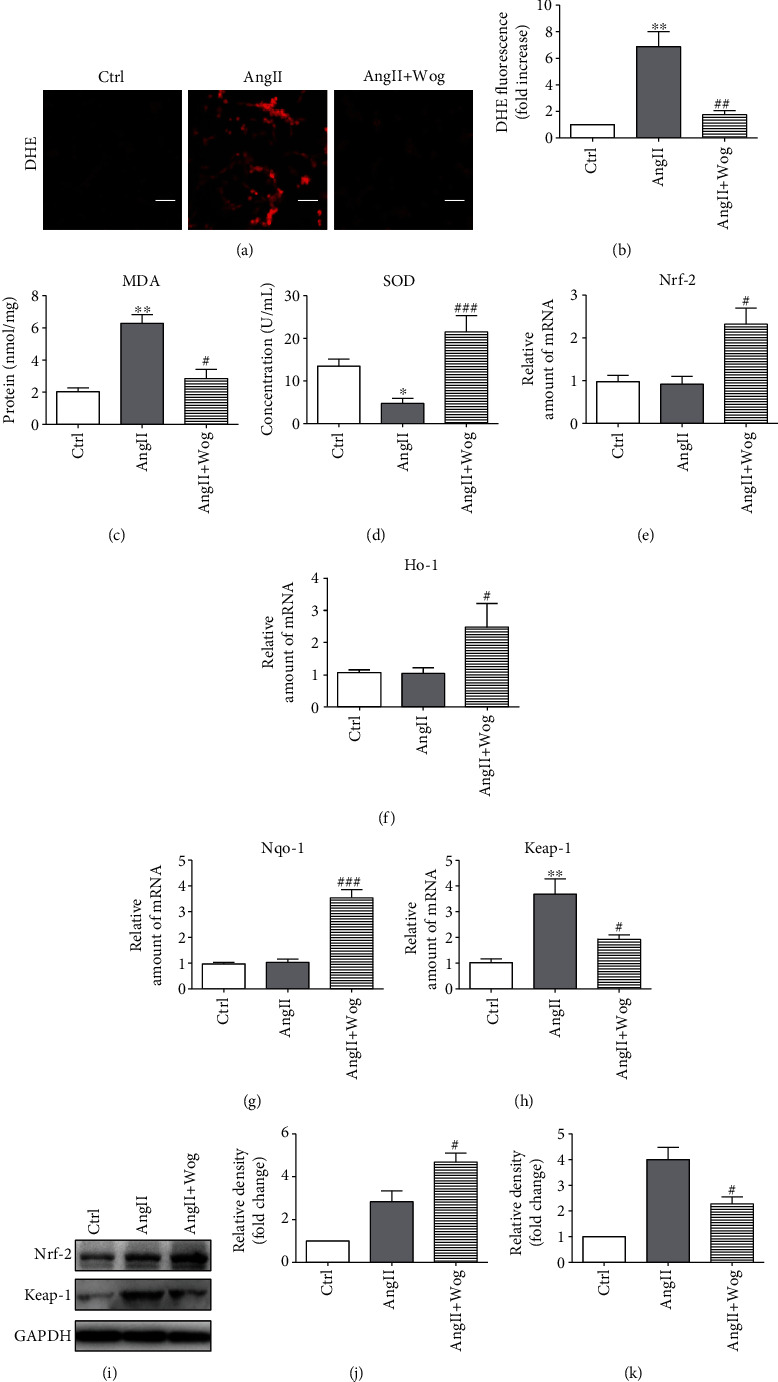
Wog inhibited oxidative stress in Ang II-induced H9C2 cells. H9C2 cells were pretreated with Wog (20 *μ*M) for 2 hours and then incubated with AngII (1 *μ*M) for the times indicated. (a, b) Representative images of DHE staining from H9C2 cells (magnification: ×200, scale bar: 20 *μ*m); (c) MDA concentration (*n* = 4); (d) SOD activity (*n* = 6); (e–h) mRNA expression of *Nrf-2*, *Keap-1*, *Ho-1*, and *Nqo-1* by RT-qPCR (*n* = 4); (i–k) Western blot analysis of Nrf-2 and Keap-1 in H9C2 cells. GAPDH was used as the loading control (*n* = 3). ^∗^*P* < 0.05, ^∗∗^*P* < 0.01, and ^∗∗∗^*P* < 0.001 compared to Ctrl; ^#^*P* < 0.05, ^##^*P* < 0.01, and ^###^*P* < 0.001 compared to AngII.

Numerous studies have confirmed that oxidative stress is linked to the onset of various cardiovascular complications, particularly cardiac hypertrophy [27–29]. Therefore, our next objective was to determine whether Wog offers protection against AngII-induced ROS generation in H9C2 cells and NRCMs. We examined the level of ROS production in cells exposed to AngII with or without Wog. Cells were pretreated with Wog for 2 h before exposure to AngII. Following the treatments, we used three assays to analyze oxidative stress in H9C2 cells and NRCMs. First, we stained the treated cells for DHE (Figures 2(a) and 2(b) and Supplementary Figure 1C and 1E). Second, we examined AngII-induced alterations in the MDA level after Wog treatment of H9C2 cells (Figure 2(c)) and NRCMs (Supplementary Figure 1F). Third, we further measured the effects of Wog on the expression of the level of total SOD (Figure 2(d) and Supplementary Figure 1G). All three assays suggested that AngII markedly increased oxidative stress levels in H9C2 cells and NRCMs. However, Wog treatment attenuated AngII-induced ROS generation and oxidative stress in H9C2 cells and NRCMs.

Next, we investigated antioxidant enzymes including Nrf-2, HO-1, and NQO-1. Kelch-like ECH-associated protein-1 (Keap-1), a negative regulator of Nrf-2, plays an important role in Nrf-2-mediated antioxidant responses. We found that compared to the AngII group, the AngII + Wog group promoted the protein and mRNA levels of Nrf-2 (Figures 2(e), 2(i), and 2(j)) and markedly decreased Keap-1 (Figures 2(h), 2(i), and 2(k)). With the activation of Nrf-2, the mRNA levels of *Ho-1* (Figure 2(f)) and *Nqo-1* (Figure 2(g)) were also upregulated in AngII-challenged cells pretreated with Wog.

### 3.3. The Cardioprotective Effects of Wog Involved Activating the Nrf-2-Mediated Antioxidant Responses

**Figure 3:**
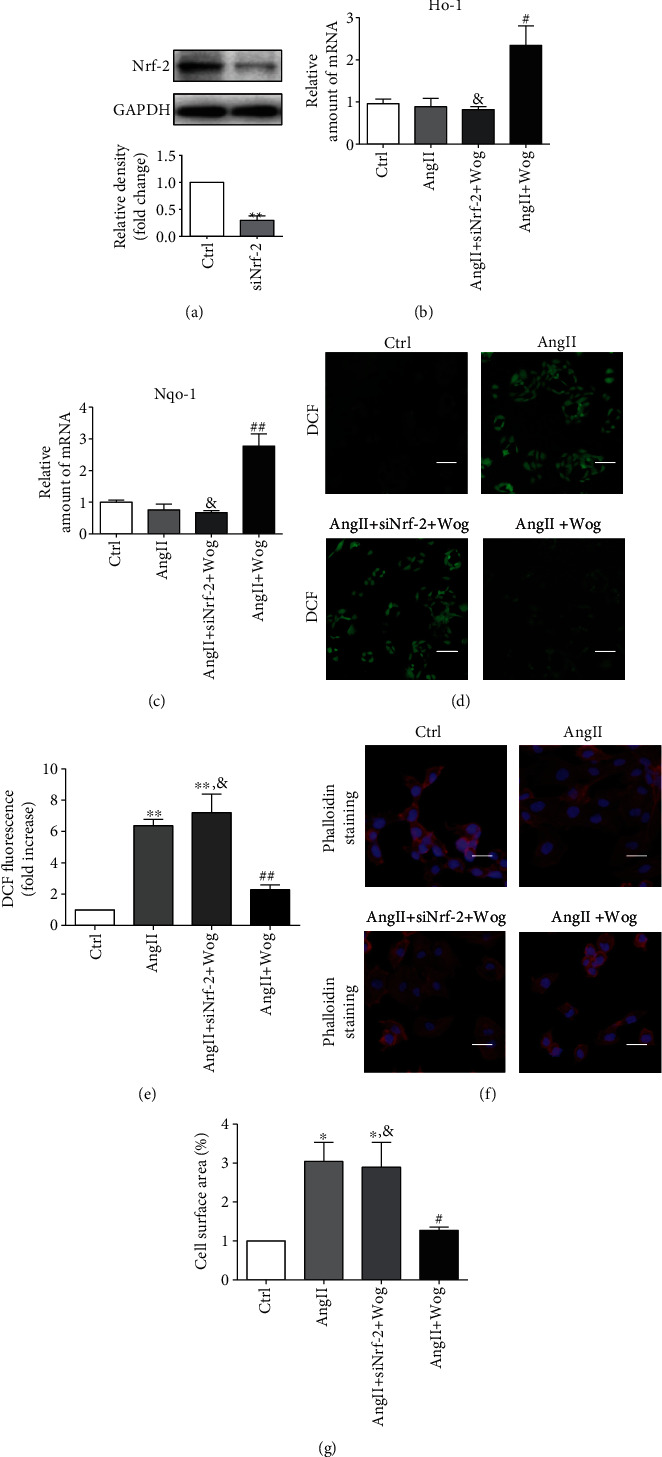
The cardioprotective effects of Wog involved in activating Nrf-2-mediated antioxidant responses. (a) Western blot analysis of Nf-2 following siRNA transfection in H9C2 cells, where GAPDH was used as the loading control (*n* = 3); (b, c) the mRNA expression of *Ho-1* and *Nqo-1* by RT-qPCR (*n* = 4); (d, e) representative images of DCF staining from H9C2 cells (magnification: ×200, scale bar: 20 *μ*m); (f, g) representative images of rhodamine-labeled phalloidin staining from H9C2 cells (magnification: ×200, scale bar: 20 *μ*m). ^∗^*P* < 0.05, ^∗∗^*P* < 0.01, and ^∗∗∗^*P* < 0.001 compared to Ctrl; ^#^*P* < 0.05, ^##^*P* < 0.01, and ^###^*P* < 0.001 compared to AngII; ^&^*P* < 0.05, ^&&^*P* < 0.01, and ^&&&^*P* < 0.001 compared to AngII + Wog.

To further confirm the role of Nrf-2 in regulating the antioxidant effects of Wog in AngII-challenged H9C2 cells, we knocked down the expression of Nrf-2 prior to AngII exposure. Compared with the Ctrl, transfection of cells with specific siRNA reduced Nrf-2 protein levels by >70% (Figure 3(a)). Wog treatment noticeably upregulated *Ho-1* (Figure 3(b)) and *Nqo-1* (Figure 3(c)) mRNA levels in AngII-challenged cells, while Wog was not able to induce these genes in Nrf-2-knockdown H9C2 cells challenged by AngII compared to the AngII + Wog group. Moreover, the treatment with siNrf-2 was unable to inhibit O_2−_ generation (Figures 3(d) and 3(e)), in AngII-stimulated Nrf2-knockdown H9C2 cells compared to the AngII + Wog group. H9C2 cell area quantization showed an evident increase in the cell area in the AngII groups compared to the Ctrl group. The Wog group could significantly reduce the cell area compared to the AngII group. However, Nrf-2 knockdown H9C2 cells were unable to inhibit the enlargement of the relative cell surface area (Figures 3(f) and 3(g)). These results showed that Wog prevented AngII-induced oxidative stress and cardiac hypertrophy in an Nrf-2-dependent mechanism.

### 3.4. Wog Prevented TAC-Induced Cardiac Hypertrophy and Dysfunction In Vivo

**Figure 4:**
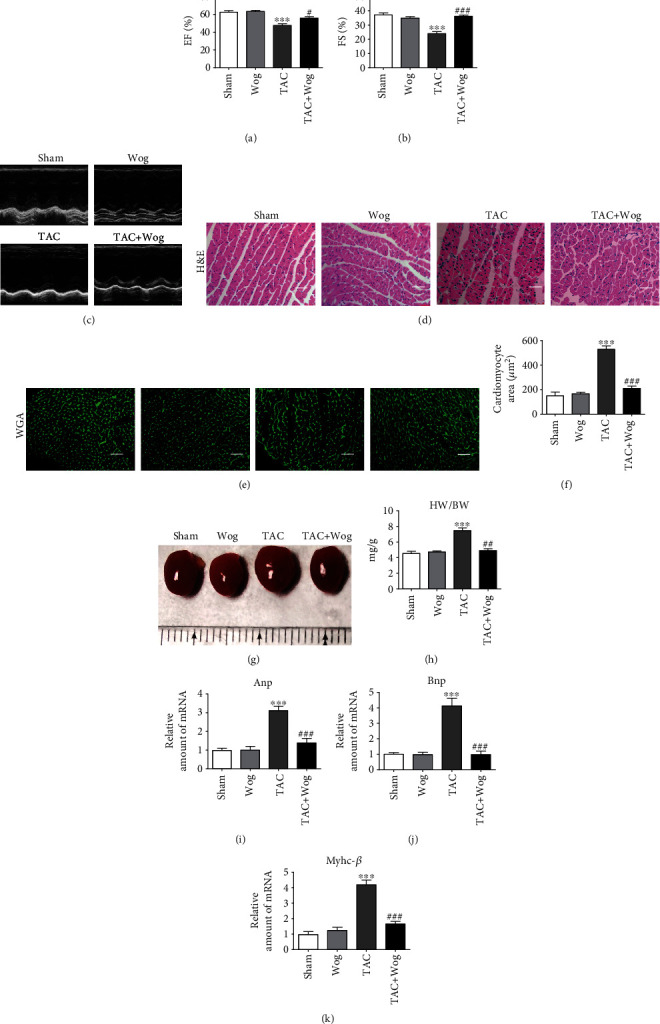
Wog prevented TAC-induced cardiac hypertrophy and dysfunction in vivo. (a, b) Echocardiographic data showed the effects of Wog on cardiac hypertrophy induced by TAC. EF: ejection fraction; FS: fractional shortening (*n* = 5); (c) representative M-mode echocardiograms from sham and TAC mice with vehicle or Wog; (d–f) histological staining of H&E and WGA of heart sections showed the inhibitory effect of Wog on cardiac hypertrophy after TAC surgery (magnification: ×200, scale bar: 20 *μ*m). Quantitative analysis of cardiomyocyte cross-sectional area (*n* = 5); (g) representative hearts of mice that underwent sham or TAC surgery, followed by single intragastric administration of Wog (10 mg/kg/day) for 8 weeks; (h) quantitative analysis of the heart weight/body weight (HW/BW) ratio (*n* = 5); (i–k) mRNA expressions for *Anp*, *Bnp*, and *Myhc-β* determined by qRT-PCR (*n* = 5). ^∗^*P* < 0.05, ^∗∗^*P* < 0.01, and ^∗∗∗^*P* < 0.001 compared to sham; ^#^*P* < 0.05, ^##^*P* < 0.01, and ^###^*P* < 0.001 compared to TAC.

Cardiac hypertrophy is an adaptive cellular response to kinds of biomechanical stresses or overload, which increase in the size and thickness of cardiac myocytes [30]. To clarify the in vivo effects of Wog in hypertrophic hearts, we established a cardiac hypertrophy model by using TAC. Eight weeks after TAC, myocardial function was assessed using echocardiography. As shown in Figures 4(a) and 4(b), compared with the TAC group, the Wog treatment (10 mg/kg/day) group showed markedly inhibited TAC-induced reduction of the ejection fraction (EF) % and fractional shortening (FS) %. Further, compared with the sham group, the TAC group showed an increase in interventricular septal dimension in diastole (IVSd), interventricular septal dimension in systole (IVSs), left ventricle end-diastolic posterior wall thickness (LVPWd), left ventricle systolic posterior wall thickness (LVPWs), left ventricular internal diameter end diastole (LVIDd), and left ventricular internal diameter end systole (LVIDs). Treatment with Wog could significantly reverse this phenomenon (Table 2). Furthermore, echocardiographic images of the left ventricle using M-mode showed that compared to the TAC group, the TAC + Wog group had recovered cardiac function (Figure 4(c)). Hematoxylin and eosin (H&E) and wheat germ agglutinin (WGA) staining of histological sections further confirmed the significant enlargement of the size of cardiomyocytes, whereas Wog suppressed these pathologic changes (Figures 4(d)–4(f)).

As shown in Figure 4(g), compared to the sham group animals, those in the TAC group showed an obvious increase in the heart size. By contrast, Wog treatment alleviated cardiac hypertrophy. Moreover, we found that compared with the sham procedure, the TAC operation significantly increased the ratios of heart weight/body weight (HW/BW); this effect was attenuated by Wog treatment (Figure 4(h)). Furthermore, compared with the sham group, the TAC group showed significant increase in mRNA expressions of cardiac hypertrophic markers of atrial natriuretic peptide (*Anp*), brain natriuretic peptide (*Bnp*), and *Myhc-β*. Wog treatment of TAC mice attenuated these parameters of myocardial hypertrophy (Figures 4(i)–4(k)).

### 3.5. The Cardioprotective Effects of Wog Involved Modulation of the Nrf-2-Mediated Antioxidant Responses In Vivo

**Figure 5:**
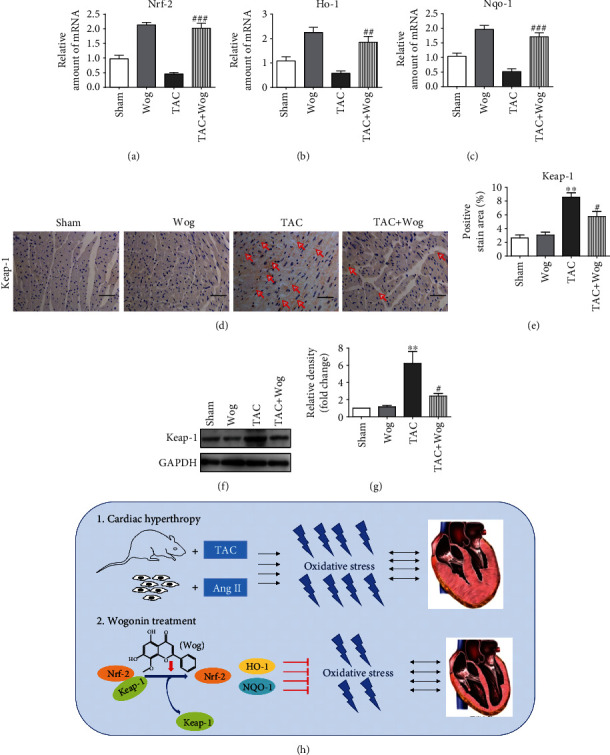
The cardioprotective effects of Wog involved in the modulation the Nrf-2-mediated antioxidant responses in vivo. (a–c) mRNA levels of *Nrf-2* target genes *Ho-1* and *Nqo-1* as determined by qRT-PCR (*n* = 5); (d, e) immunohistochemical staining of heart tissues for Keap-1, the positive region with the red arrow; (f, g) heart lysates were probed for Keap-1 levels with Western blotting. GAPDH was used as the loading control (*n* = 3); (h) schematic diagram summarizes the molecular mechanisms by which Wog ameliorates cardiac hypertrophy by inhibiting oxidative stress. Wog as a promising natural agent, which protects against cardiac hypertrophy by activating the Nrf-2-mediated antioxidant responses. ^∗^*P* < 0.05, ^∗∗^*P* < 0.01, and ^∗∗∗^*P* < 0.001 compared to sham; ^#^*P* < 0.05, ^##^*P* < 0.01, and ^###^*P* < 0.001 compared to TAC.

Consistent with the results from the AngII model, the mRNA expression levels of *Nrf-2*, *Ho-1*, and *Nqo-1* of the in vivo model were measured by RT-qPCR. As shown in Figures 5(c)–5(c), the mRNA levels of *Nrf-2*, *Ho-1*, and *Nqo-1* were also upregulated in the TAC + Wog group compared with the TAC group. Next, we assessed the expression of Keap-1 in TAC-challenged heart tissue and determined whether Wog was cardioprotective. Our data showed that Keap-1 staining and protein concentration significantly increased in the TAC group, but was restored to baseline with Wog treatment (Figures 5(d)–5(g)). Based on the above results, we suggested that Wog as a promising natural agent, which protects against cardiac hypertrophy by activating the Nrf-2-mediated antioxidant responses. (Figure 5(h)).

## 4. Discussion

In this study, the in vitro model of cardiac hypertrophy was established by stimulating H9C2 cells with AngII, which led to significant cardiomyocyte hypertrophy and increased oxidative stress levels. Wog treatment significantly inhibited oxidative stress and ameliorated cardiac hypertrophy. Additionally, the in vivo results in C57BL/6 mice induced by TAC were entirely consistent with the results obtained in in vitro experiments. We found that these antioxidative effects of Wog were mediated through the Keap-1/Nrf-2 pathway. Therefore, Wog has potential therapeutic value in myocardial hypertrophy and the Keap-1/Nrf-2 pathway might serve as a therapeutic target in the treatment of cardiac hypertrophy.

Cardiac hypertrophy, one of the initial disorders in the cardiovascular system, is known to induce heart failure [31]. Cardiac hypertrophy, including physiological and pathological hypertrophy, is usually identified by an increase in cell size [32]. Despite this understanding of the pathogenesis, treatment options for cardiac hypertrophy are ineffective and limited. Therefore, new methods for the treatment of cardiac hypertrophy, remodeling, and heart failure still need to be developed. Our results showed that Wog significantly attenuated AngII-induced cardiomyocyte hypertrophy and oxidative stress in H9C2 cells (Figures 1 and 2). We established an in vivo model of cardiac hypertrophy using TAC and found that Wog treatment could significantly prevent cardiac hypertrophy and dysfunction (Figure 4). Furthermore, Wog inhibited TAC-induced oxidative stress and induced the expression of antioxidant response proteins (Figures 5(a)–5(c)), in agreement with the results from the in vitro model. Thus, we could conclude that these antioxidant effects of Wog were the key to preventing and treating cardiac hypertrophy.

It is known that Nrf-2 is a ubiquitous master transcription factor that upregulates some antioxidative enzymes such as HO-1, NQO-1, SOD, and GSH-Px [33]. This pathway is proven to be related with many cardiovascular diseases including hypertrophy. Importantly, Nrf-2 is considered an important potential target in the treatment of cardiac hypertrophy and heart failure [34]. To confirm that Wog protects against cardiac hypertrophy by activating the Nrf-2-mediated antioxidant responses, we use specific siRNA for *Nrf-2*. We detected that Wog could not induce HO-1 or NQO-1 or inhibit ROS generation and hypertrophy in AngII-stimulated Nrf-2-knockdown H9C2 cells (Figure 3). Keap-1 is an endogenous inhibitor and regulator of Nrf-2. Normally, Nrf-2 is present in the cytoplasm by binding to its inhibitor Keap-1. Under oxidative stress conditions, Nrf-2 separates from Keap-1 and is transferred to the nucleus to promote expression of its downstream antioxidant genes such as *Ho-1* and *Nqo-1*. We further determined whether Wog could activate the Nrf-2 antioxidant pathway by modulating the Keap-1/Nrf-2 pathway. As shown in Figures 5(d)–5(g), Keap-1 staining and protein concentration were significantly increased in the TAC group and were restored with Wog treatment. Wog might modulate the Keap-1/Nrf-2 pathway and promote the expression of antioxidant response mediated by Nrf-2. Thus, we can conclude that the cardioprotective effects of Wog involve modulation of the Keap-1/Nrf-2 pathway.

## 5. Conclusion

Collectively, these results suggested that Wog, a major flavonoid of *Scutellaria baicalensis Georgi*, protects against cardiac hypertrophy by activating the Nrf-2 pathway. These findings provide support for future research and the potential use of Wog to improve treatment for cardiac hypertrophy (Figure 5(h)). Although our results are promising, our study has some limitations. Further studies are needed to verify that Wog specifically targets Nrf-2 to promote the expression of its downstream antioxidant genes *Ho-1* and *Nqo-1* and attenuates oxidative stress in cardiac hypertrophy.

## Figures and Tables

**Table 1:** Sequences of primers for real-time qPCR assay used in the study.

Gene	Species	Forward	Reverse
*Nrf-2*	Rat	GCATTTCGCTGAACACAA	CTCTTCCATTTCCGAGTCA
*Ho-1*	Rat	AGAGTTTCTTCGCCAGAGG	GAGTGTGAGGACCCATCG
*Nqo-1*	Rat	GCTTTCAGTTTTCGCCTTT	CCTCGTTCATTTTGCTGTC
*Keap-1*	Rat	GGCAGAAGAGGCAGCAG	AGGGGCTATGACAGAAGGG
*Anp*	Rat	GGGCTCCTTCTCCATCACC	CTCCAATCCTGTCAATCCTACC
*Bnp*	Rat	CCTAAAACAACCTCAGCCCGT	TTCCGGATCCAGGAGAGACTT
*Myhc-β*	Rat	GAGGAGAGGGCGGACATT	ACTCTTCATTCAGGCCCTTG
*β-Actin*	Rat	AAGTCCCTCACCCTCCCAAAAG	AAGCAATGCTGTCACCTTCCC

**Table 2:** Biometric and echocardiographic parameters of mice in each group.

Parameter	Sham (*n* = 5)	Wog (*n* = 5)	TAC (*n* = 5)	TAC + Wog (*n* = 5)
IVSd (mm)	0.727 ± 0.078	0.748 ± 0.054	0.913 ± 0.083^∗^	0.772 ± 0.041^#^
IVSs (mm)	1.045 ± 0.147	1.069 ± 0.051	1.336 ± 0.05^∗∗^	1.101 ± 0.084^##^
LVPWd (mm)	0.734 ± 0.065	0.707 ± 0.052	0.840 ± 0.04^∗^	0.733 ± 0.032^##^
LVPWs (mm)	1.069 ± 0.048	1.083 ± 0.120	1.222 ± 0.098^∗^	1.056 ± 0.103^#^
LVIDd (mm)	3.087 ± 0.165	3.071 ± 0.101	4.289 ± 0.256^∗∗∗^	3.328 ± 0.142^###^
LVIDs (mm)	1.878 ± 0.181	1.902 ± 0.222	3.307 ± 0.184^∗∗∗^	2.438 ± 0.111^###^

Interventricular septal dimension in diastole (IVSd), interventricular septal dimension in systole (IVSs), left ventricle end-diastolic posterior wall thickness (LVPWd), left ventricle systolic posterior wall thickness (LVPWs), left ventricular internal diameter end diastole (LVIDd), left ventricular internal diameter end systole (LVIDs). Values shown as mean ± SEM. ^∗^*P* < 0.05, ^∗∗^*P* < 0.01, and ^∗∗∗^*P* < 0.001 compared with sham; ^#^*P* < 0.05, ^##^*P* < 0.01, and ^###^*P* < 0.001 compared with TAC. *P* values by one-way ANOVA followed by Tukey's post hoc test are indicated.

## Data Availability

The datasets used and/or analyzed during the current study are available from the corresponding author upon reasonable request. All data generated or analyzed during this study are included in this published article.

## Abbreviations

Wog: Wogonin
AngII: Angiotensin II
NRCMs: Neonatal rat cardiomyocytes
TAC: Transverse aortic constriction
Nrf-2: Nuclear factor- (erythroid-derived 2-) like 2
HO-1: Heme oxygenase-1
NQO-1: NADPH quinine oxidoreductase-1
H9C2: Rat myocardial cells
Keap-1: Kelch-like ECH-associated protein-1
MDA: Malondialdehyde
SOD: Superoxide dismutase
DHE: Dihydroethidium
BCA: Bicinchoninic acid
SDS-PAGE: Sodium dodecyl sulfate-polyacrylamide gel electrophoresis
PVDF: Polyvinylidene difluoride
BSA: Bovine serum albumin
EF: Ejection fraction
FS: Fractional shortening
IVSd: Interventricular septal dimension in diastole
IVSs: Interventricular septal dimension in systole
LVPWd: Left ventricle end-diastolic posterior wall thickness
LVPWs: Left ventricle systolic posterior wall thickness
LVIDd: Left ventricular internal diameter end diastole
LVIDs: Left ventricular internal diameter end systole
H&E: Hematoxylin and eosin
WGA: Wheat germ agglutinin
HW/BW: Heart weight/body weight
ANP: Atrial natriuretic peptide
BNP: Brain natriuretic peptide
MyHC-*β*: Myosin heavy chain-beta.

## Supplementary Materials

Supplementary Materials As shown in supplementary Figure 1, Wog prevented AngII-induced hypertrophy and oxidative stress in NRCMs.
