# Corrigendum to “Wogonin Inhibits Cardiac Hypertrophy by Activating Nrf-2-Mediated Antioxidant Responses”

**DOI:** 10.1155/cdr/9864046

**Published:** 2025-07-31

X. Shi, B. Zhang, Z. Chu, et al., “Wogonin Inhibits Cardiac Hypertrophy by Activating Nrf-2-Mediated Antioxidant Responses”, *Cardiovascular Therapeutics* 2021 (2021): 9995342, https://doi.org/10.1155/2021/9995342

In the article, there is an error in Figure 4e due to the incorrect selection of images during manuscript preparation. The correct Figure 4 is shown below:

The authors apologize for this error.

## Figures and Tables

**Figure 1:**
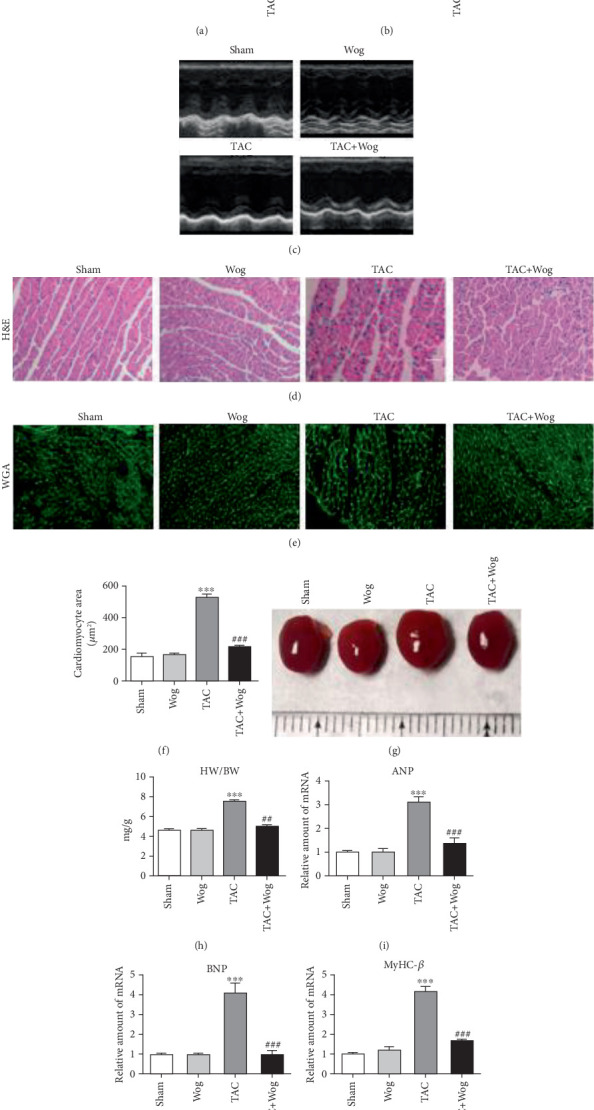
Wog prevented TAC-induced cardiac hypertrophy and dysfunction in vivo. (a, b) Echocardiographic data showed the effects of Wog on cardiac hypertrophy induced by TAC. EF: ejection fraction; FS: fractional shortening (*n* = 5); (c) representative M-mode echocardiograms from sham and TAC mice with vehicle or Wog; (d–f) histological staining of H&E and WGA of heart sections showed the inhibitory effect of Wog on cardiac hypertrophy after TAC surgery (magnification: ×200, scale bar: 20 *μ*m). Quantitative analysis of cardiomyocyte cross-sectional area (*n* = 5); (g) representative hearts of mice that underwent sham or TAC surgery, followed by single intragastric administration of Wog (10 mg/kg/day) for 8 weeks; (h) quantitative analysis of the heart weight/body weight (HW/BW) ratio (*n* = 5); (i–k) mRNA expressions for Anp, Bnp, and Myhc-*β* determined by qRT-PCR (*n* = 5). ⁣^∗^*p* < 0.05, ⁣^∗∗^*p* < 0.01, and ⁣^∗∗∗^*p* < 0.01 compared to sham; ^#^*p* < 0.05, ^##^*p* < 0.01, and ^###^*p* < 0.01 compared to TAC.

