# Supplementary material for: Wogonin Inhibits Cardiac Hypertrophy by Activating Nrf-2-Mediated Antioxidant Responses
Source: Cardiovasc Ther. 2021 Jul 1;2021:9995342. doi: 10.1155/2021/9995342 (PMC8266446; doi:10.1155/2021/9995342)
Supplement: Supplementary Materials — As shown in supplementary Figure 1, Wog prevented AngII-induced hypertrophy and oxidative stress in NRCMs. [file 9995342.f1.zip › Supplementary Figure-Wog (2).docx]

**Wogonin inhibits** **cardiac hypertrophy** **by activating Nrf-2-mediated antioxidant responses**

Xiaowen Shi^#^, Bin Zhang^#^, Zhenliang Chu^#^, Bingjiang Han, Xueping Zhang, Ping Huang, Jibo Han^*^

Department of Cardiology, The Second Affiliated Hospital of Jiaxing University, Jiaxing, Zhejiang 314000, China

#These authors contributed equally to this work

Supplementary Fig. 1


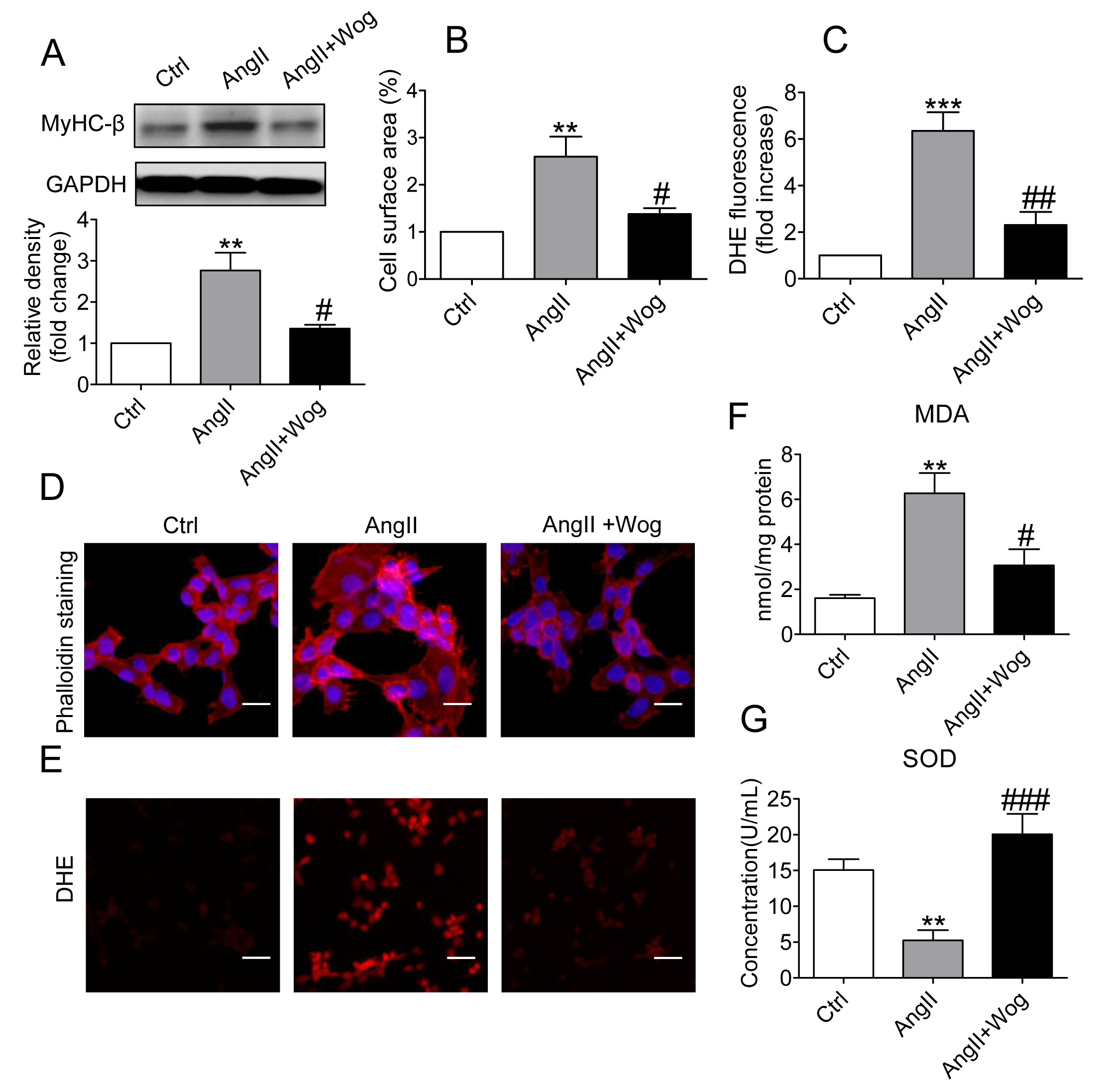


**Supplementary Fig. 1**

Notes: NRCMs were pretreated with Wog (20 µM) for 2 hour and then incubated with AngII (1 µM) for the times indicated. (A) Western blot analysis of MyHC-β in NRCMs (n=3); (B, D) Representative images of rhodamine-labeled phalloidin staining from NRCMs (magnification: ×200, scale bar: 20 µm); (C, E) Representative images of DHE staining from NRCMs (magnification: ×200, scale bar: 20 µm); (F) MDA concentration (n=4); (G) SOD activity (n=6).
