# Supplementary figures and images for: Wogonin Inhibits Cardiac Hypertrophy by Activating Nrf-2-Mediated Antioxidant Responses
Source: Cardiovasc Ther. 2021 Jul 1;2021:9995342. doi: 10.1155/2021/9995342 (PMC8266446; doi:10.1155/2021/9995342)

## Slide 1
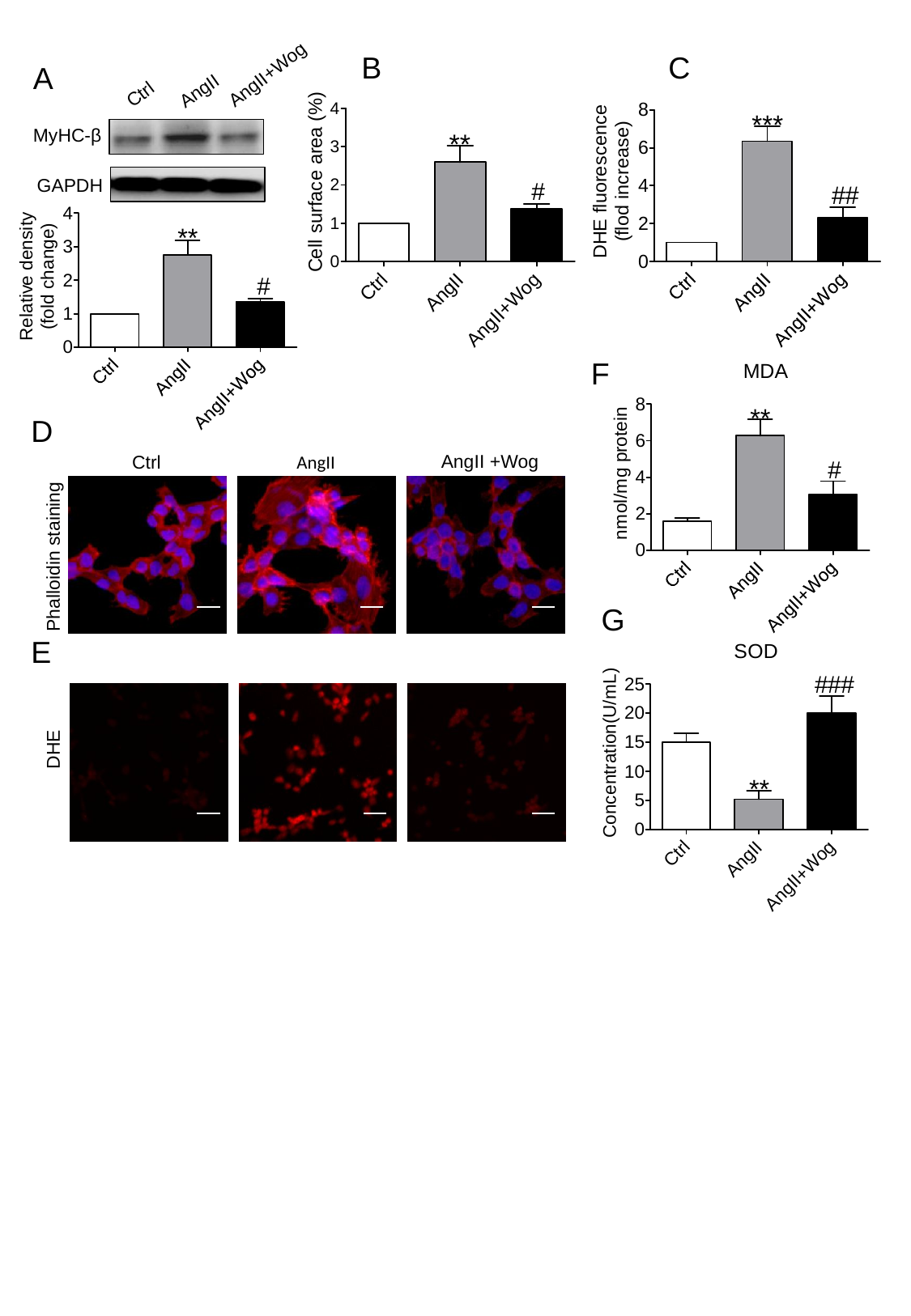

AngII+Wog
Ctrl
AngII
MyHC-β
GAPDH
B
C
A
F
D
AngII +Wog
Ctrl
AngII
Phalloidin staining
G
E
DHE

Supplement: Supplementary Materials — As shown in supplementary Figure 1, Wog prevented AngII-induced hypertrophy and oxidative stress in NRCMs. [file 9995342.f1.zip › Supplementary Fig. 1.pptx]
